# Direct Oral Anticoagulants versus Vitamin K Antagonists in Patients Aged 80 Years and Older

**DOI:** 10.3390/ijerph18094443

**Published:** 2021-04-22

**Authors:** Patrick Manckoundia, Gilles Nuemi, Arthur Hacquin, Didier Menu, Clémentine Rosay, Jérémie Vovelle, Valentine Nuss, Camille Baudin-Senegas, Jérémy Barben, Alain Putot

**Affiliations:** 1 “Pôle Personnes Âgées”, Hospital of Champmaillot, University Hospital, 21079 Dijon, France; arthur.hacquin@chu-dijon.fr (A.H.); rosayclm@gmail.com (C.R.); jeremie.vovelle@chu-dijon.fr (J.V.); valentine.nuss@chu-dijon.fr (V.N.); camille.senegas@chu-dijon.fr (C.B.-S.); jeremy.barben@chu-dijon.fr (J.B.); alain.putot@chu-dijon.fr (A.P.); 2 INSERM U-1093, Cognition, Action and Sensorimotor Plasticity, University of Burgundy Franche-Comté, 21000 Dijon, France; 3 Department of Medical Information, University Hospital, 21079 Dijon, France; gilles.nuemi@chu-dijon.fr; 4 “Mutualité Sociale Agricole” of Burgundy, 21079 Dijon, France; menu.didier@bourgogne.msa.fr; 5 Physiopathologie et Epidémiologie Cérébro-Cardiovasculaires (PEC2), University of Burgundy and Franche Comté, 21000 Dijon, France

**Keywords:** aged 80 and over, anticoagulant, direct oral anticoagulants, vitamin K antagonists

## Abstract

The effectiveness of direct oral anticoagulants (DOAC) is non-inferior to vitamin K antagonists (VKA) to treat atrial fibrillation and venous thromboembolism (VTE). In this cross-sectional study, we compared older persons taking DOACs to those taking VKAs. We included ambulatory individuals ≥80 years, affiliated to Mutualité Sociale Agricole of Burgundy, who were refunded for a medical prescription in September 2017. The demographic conditions, registered chronic diseases (RCD), and number and types of prescribed drugs were compared in the DOAC group and VKA group. Of the 3190 included individuals, 1279 (40%) were prescribed DOACs and 1911 (60%) VKAs. Individuals taking VKAs were older than those taking DOACs (87.11 vs. 86.35 years). In the DOAC group, there were more women (51.92% vs. 48.25%) (*p* = 0.043), less RCD (89.60% vs. 92.73%) (*p* = 0.002), less VTE (1.80% vs. 6.59%), less severe heart failure (58.09% vs. 67.87%), less severe hypertension (18.22% vs. 23.60%), less severe kidney diseases (1.49% vs. 3.82%), and fewer drugs per prescription (6.15 vs. 6.66) (*p* < 0.01 for all). The DOAC group were also less likely to be taking angiotensin receptor blockers (10.79% vs. 13.97%), furosemide (40.81% vs. 49.66%) or digoxin (10.32% vs. 13.66%) than the VKA group (*p* = 0.009, *p* < 0.001, and *p* = 0.005). DOACs were less prescribed than VKAs. Individuals taking VKAs were older and had more severe comorbidities and more drugs per prescription than those taking DOACs.

## 1. Introduction

The number of healthy older persons and frail older individuals is increasing worldwide due to the increase in life expectancy [1]. Furthermore, the frequency of cardiovascular, cerebrovascular, and vascular diseases increases with advancing age as a result of the independent effect of aging, and because the number of risk factors increases with age [2]. Finally, cardiovascular and cerebrovascular events in older persons result in a higher rate of long-term disability and dependence [3]. Thus, it is very important to provide optimal treatment of cardiovascular and cerebrovascular diseases in order to prevent their harmful consequences. Primary and secondary prevention, as well as curative treatment of the vast majority of cardiovascular, cerebrovascular, and peripheral vascular events, such as atrial fibrillation (AF) and venous thromboembolism (VTE), require antithrombotic therapy [4,5].

Of the two main oral anticoagulants, vitamin K antagonists (VKA) are the oldest on the market. VKAs have been used since the 1940s when warfarin was approved for the treatment of VTE [6]. The two other main VKAs used in France are fluindione and acenocoumarol. Direct oral anticoagulants (DOAC) were developed more recently as an alternative to VKAs. In 2008, dabigatran, a direct thrombin-inhibitor, became the first DOAC approved in the European Union for stroke prevention in relation to AF [7]. The three other DOACs are rivaroxaban, apixaban, and edoxaban, which are factor Xa-inhibitors.

Both DOACs and VKAs are indicated: (1) to prevent stroke in AF, (2) in curative treatment of VTE, including pulmonary embolism, and (3) in prevention of VTE recurrence. However, DOACs cannot be used in valvular AF [8,9]. Another specific indication for DOACs is the prevention of VTE after surgery for total hip or knee prosthesis [8]. In addition, rivaroxaban is indicated in prevention of atherothrombotic events after acute coronary syndrome or in case of stable coronary or peripheral artery disease [8]. As concerns VKAs, their specific indication is the prevention of thromboembolic complications in other heart rhythm disorders (atrial flutter, atrial tachycardia), mitral valve disease, heart valve prosthesis, or myocardial infarction with embolic risk (for example mural thrombus or severe left ventricular dysfunction) [9]. The effectiveness of DOACs is at least equal to that of VKAs for treatment of AF and VTE [10,11]. As concerns bleeding risk, a meta-analysis showed a decrease in intracranial hemorrhages and an increase in gastrointestinal bleedings with DOACs compared to VKAs [12]. In addition, while VKA treatment requires monitoring for anticoagulant activity by an assay of the international-normalized-ratio (INR), DOACs do not [13]. On the contrary, DOACs must be used with caution in patients with kidney failure because of the increased risk of bleeding [14]. The latest guidelines from the European Society of Cardiology (ESC) and European Heart Rhythm Association recommend using DOACs rather than VKAs when there is a need for oral anticoagulation in patients with diagnosed AF and with no contraindication to DOACs [4,15].

In clinical practice in France, our observations suggest that physicians can be reluctant to prescribe new molecules in older individuals and that some physicians prefer to gain experience prescribing novel treatments in younger adults before prescribing them in the elderly. We, therefore, conducted this study to compare the prescription rates of DOACs and VKAs in ambulatory older adults. In addition, we compared the age, sex, and medical characteristics of older persons treated with DOACs to those treated with VKAs in order to try to identify factors associated with the prescription of one anticoagulant or the other.

## 2. Methods

### 2.1. Study Design

This cross-sectional study used data collected between the 1st and 30th September 2017 from the existing database of a French regional agricultural health insurance agency (Mutualité Sociale Agricole de Bourgogne). This study was conducted in accordance with the Declaration of Helsinki and French national standards.

### 2.2. Subjects

The population consisted of all Mutualité Sociale Agricole de Bourgogne-affiliated individuals aged 80 years and older living in Burgundy who were refunded for a treatment prescribed during an ambulatory medical consultation between the 1st and 30th September 2017. In total, two groups were constituted, one composed of subjects with a direct oral anticoagulant prescription (DOAC group) and the other composed of subjects with VKA prescription (VKA group).

The Ethics Committee of our institution was consulted (2018-1002-PM). It approved this study which did not affect patient management.

### 2.3. Collected Data

For each subject, age (years), sex, the duration of the prescription (to determine if it was a novel or refill prescription) (≥3 months), and the medical specialty of the prescribing physician were collected. For a given subject, DOAC or VKA prescription was considered novel if (1) it was made within three months preceding the date of inclusion, and (2) if no DOAC or VKA prescription was found more than three months before inclusion. This three-month period was chosen because in France, the maximum period of validity of a prescription for a drug from “list 1”, to which DOACs and VKAs belong, is three months. List 1 includes drugs that can be toxic under normal conditions of use. These drugs can only be dispensed on presentation of a medical prescription and for the duration specified on the prescription. In addition, we recorded registered chronic diseases (RCD), according the International Classification of Diseases, 10th revision [16]. A RCD is a disease whose severity and chronic nature require prolonged and particularly expensive treatment, and which confers the right to the total coverage of health costs by the French public health insurance system, except for non-exempting illnesses (i.e., medical conditions requiring an interruption of work or care for a longer period than 6 months and for which the costs are not fully covered by that institution). Among RCDs, there are disabling stroke, bone marrow failure and other chronic cytopenia, chronic arterial occlusive diseases with ischemic manifestations, severe heart failure, or heart rhythm disorders (including recurrent paroxysmal, persistent or permanent AF), active chronic liver diseases and cirrhosis, diabetes types 1 and 2, neuropathy or myopathy, and epilepsy, severe hypertension, coronary artery disease, severe chronic respiratory failure, Alzheimer’s disease and other dementias, Parkinson disease, severe chronic nephropathy and primitive nephrotic syndrome, vasculitis or systemic lupus erythematosus or systemic scleroderma, rheumatoid arthritis, psychotic disorders, cancers or hematologic malignancy, illnesses not on the list, polymorbidity, and non-exempting illnesses. Certain medical conditions must be severe to be recognized as RCDs. Severe heart failure is defined by the association of (1) symptoms of heart failure (at rest or on exercise) and objective evidence of systolic heart dysfunction at rest with a left ventricular ejection fraction < 40%, for chronic systolic heart failure or (2) symptoms of heart failure (at rest or on exercise), objective cardiac dysfunction sign(s) at rest on ECG or paraclinical exams with preserved or moderately impaired systolic function (left ventricular ejection fraction > 40%) and a pharmacological response to heart failure treatment, for chronic heart failure with preserved ejection fraction. Hypertension is severe in case of (1) blood pressure ≥ 180/110 mmHg; or (2) blood pressure < 180/110 mmHg but >140/90 mmHg measured several times separated by several weeks, and associated with at least one of signs of the following organic impacts: left ventricular hypertrophy and myocardial ischemia, coronary insufficiency, microalbuminuria at 30 mg/day or 20 mg/L; kidney failure (glomerular filtration rate < 60 mL/min) or proteinuria > 500 mg/day, transient ischemic attack or stroke, hemorrhages or exudates or papillary edema on fundus, arterial occlusive disease of the lower limbs and aortoiliac; or (3) continuous prescription for 3 months, of at least 3 classes of antihypertensive drugs and each at the optimal daily dose. Severe chronic respiratory failure is defined by arterial partial pressure of oxygen < 60 mmHg and partial pressure of carbon dioxide > 50 mmHg at two measurements separated by at least 15 days or forced expiratory volume in 1 s < 50% at two measurements separated by at least 1 month. Severe chronic nephropathy include glomerular, interstitial, vascular, tubular, or hereditary renal diseases of a chronic nature with at least one of the following criteria: (1) glomerular filtration rate < 60 mL/min, at two measurements separated by at least 3 months; (2) permanent proteinuria > 1 g/24 h/1.73 m²; (3) permanent arterial hypertension (≥130/80 mmHg) requiring long-term drug treatment; (4) phosphocalcic, acid-base or electrolyte metabolic disorders, or anemia requiring treatment and biological monitoring; and (5) chronic uropathy requiring continued care and monitoring. Illnesses not on the list include active or disabling forms of serious diseases, not individually named, requiring expensive treatment for duration of more than 6 months [17]. RCDs are declared to health insurance by the patient’s general practitioner (GP) or medical specialist. We also collected the number of drugs per prescription, concomitant cardiovascular medications including anti-thrombotics, beta-blockers, alpha-blockers, angiotensin-conversion-enzyme inhibitors, angiotensin receptor blockers, calcium-channel-blockers, nitrate derivatives and other vasodilators, diuretics, cardiac glycosides, other antiarrhythmic drugs (i.e., classes Ia, Ic, and III antiarrhythmic drugs according to Vaughan Williams classification [18]), and hypolipidemic drugs. Data regarding the prescription of biological tests were collected. They included complete blood cell count, serum creatinine, blood urea nitrogen, aspartate aminotransferase and alanine aminotransferase rate, γ-glutamyltranspeptidase rate, alkaline phosphatase rate, INR, prothrombin time and activated partial thromboplastin time. Finally, we deduced from the declared RCD whether the indication(s) for oral anticoagulation was AF or VTE. Because our aim was to compare the DOAC and VKA groups, we limited the collection of the indication(s) for oral anticoagulation to only these two events, i.e., AF and VTE, because they are the only common indications for DOACs and VKAs.

### 2.4. Statistical Analysis

Quantitative variables were described as means and standard deviations, while categorical variables were described as numbers and percentages.

The two groups (DOAC and VKA) were compared in terms of mean age, age range, sex, mean number of RCDs, mean number of drugs per prescription, existence of one or more RCD, anticoagulant prescription duration (novel prescription or refill), prescriber specialty, rates of AF and VTE, frequency of selected RCD, and cardiovascular medications. In bivariate analysis, data were compared using the chi-squared test or the Fisher test for categorical variables, and the analysis of variance for quantitative variables. Statistical significance was set at *p* < 0.05. In order to study the association between the type of prescribed anticoagulant and each parameter, we performed a bivariate analysis using logistic regression, with the calculation of odds ratios (OR) and 95% confidence intervals (95% CI). Then, a multivariate analysis using stepwise logistic regression was performed. The multivariate analysis included variables for which at least one of the sizes of the 2 groups was greater than 10 and, otherwise, responding to multicollinearity.

R Core Team (2019) software (R Foundation for Statistical Computing, Vienna, Austria) was used to conduct all statistical analyses [19].

## 3. Results

In the studied population, 3190 older adults with a mean age (years) of 86.81 ± 4.40 (range 80 to 103) filled a prescription for anticoagulants. 50.28% were men and 49.71% were women. The DOAC group included 1279 individuals (40%) and the VKA group included 1911 individuals (60%).

Table 1 shows mean age, age ranges, sex, the existence of one or more RCD, anticoagulant prescription duration, medical specialty of the prescribing physician, rates of AF and VTE, mean number of RCD, and mean number of drugs per prescription in the DOAC group and VKA group. Individuals with VKAs were significantly older than those with DOACs, respectively, 87.11 ± 4.44 (range 80 to 103) and 86.35 ± 4.29 (range 80 to 99) (*p* < 0.001). There were significantly more women in the DOAC group than in the VKA group, 51.92% vs. 48.25%, respectively (*p* = 0.043). The mean number of RCDs was significantly lower in the DOAC group than in the VKA group, 1.80 ± 1.17 and 2.07 ± 1.22, respectively (*p* < 0.001). It was the same for the mean number of drugs per prescription, 6.15 ± 2.84 and 6.66 ± 2.86, respectively (*p* < 0.001). There were significantly fewer individuals with ≥1 RCD in the DOAC group than in the VKA group, 89.60% vs. 92.73%, respectively (*p* = 0.002). There were more refill prescriptions than novel prescriptions in both groups, with significantly less novel prescriptions in the DOAC group than in the VKA group, 7.35% and 11.62%, respectively (*p* < 0.001). The prescriber was most often the GP in both groups, but there were significantly less GP prescribers in the DOAC group than in the VKA group, 90.70% vs. 94.71%, respectively (*p* < 0.001). The rate of individuals with AF was similar in the two groups (41.36% and 44.22%, *p* = 0.11), while the rate of individuals with VTE was significantly lower in the DOAC group than in the VKA group, 1.80% and 6.59%, respectively (*p* < 0.001).

As concerns the types of DOACs used, apixaban (*N* = 561, 43.86%) was the most prescribed DOAC, followed by rivaroxaban (*N* = 481, 37.61%) and dabigatran (*N* = 237, 18.53%). Edoxaban was not prescribed in our study because it is not marketed in France. In the VKA group, fluindione (*N* = 1162, 60.81%) was the most prescribed VKA, followed by warfarin (*N* = 679, 35.53%) and acenocoumarol (*N* = 70, 3.66%).

Table 2 compares RCDs in the DOAC group and the VKA group using bivariate analysis by logistic regression. The patients in the DOAC group had significantly less of the following: severe heart failure or heart rhythm disorders, severe hypertension, severe chronic respiratory failure, severe kidney diseases and illnesses not on the list (all *p* < 0.001, expect for severe chronic respiratory failure *p* = 0.006). There were no significant differences between the two groups for active chronic liver diseases and cirrhosis (2 subjects in each group, *p* = 1, OR (95% CI) = 0.67 (0.09–4.75)) or other RCDs. Table 2 also presents the comparison of prescriptions for cardiovascular medications in the DOAC group and the VKA group using bivariate analysis by logistic regression. Angiotensin receptor blockers, furosemide, and digoxin were significantly less prescribed in the DOAC group than in the VKA group (*p* = 0.009, *p* < 0.001, and *p* = 0.005, respectively), while the other antiarrhythmic drugs were less prescribed in the VKA group (*p* = 0.004). There were no significant differences for the other drugs (platelet aggregation inhibitors, beta-blockers, central, and peripheral alpha-blockers, angiotensin-conversion-enzyme inhibitors, calcium-channel-blockers, nitrate derivatives and other vasodilators, thiazide diuretics, spironolactone, and all types of hypolipidemic drugs).

As shown in Table 3, refill prescriptions, AF, and the prescription of beta-blockers and other antiarrhythmic drugs were significant determinants for DOAC vs. VKA prescription in multivariate analysis. On the contrary, age, number of drugs per prescription, male sex, GP prescribers, VTE, severe heart failure or heart rhythm disorders, severe hypertension, severe kidney failure, and the presence of angiotensin-receptor-blocker were significant determining factors for VKA prescription vs. DOAC prescription.

## 4. Discussion

The interest of our study is that it analyzes the prescribing practices for oral anticoagulants in an elderly population. In addition, our study was performed on real-life data using a health insurance agency database. Indeed, we found it relevant to compare DOAC and VKA prescriptions a few years after DOACs were approved for the marketplace.

Our population of 3190 individuals consuming anticoagulants was elderly (mean age 86.81). There were slightly more men (50.28%) than women, which is surprising considering the longer life expectancy in women, especially in France [20]. However, the difference in the distribution by sex, less than 0.6%, was minor. We can, therefore, consider that there were as many women as men treated with oral anticoagulants (DOACs and VKAs combined).

Although there are several studies comparing DOACs with VKAs, few of them focused on elderly persons, and they mainly focused on effectiveness and safety, including adverse events and adherence [21,22,23].

We found that people treated with VKAs were older than those who were treated with DOACs. This was confirmed in a multivariate analysis which showed that age was an independent factor associated with VKA prescription. However, this result must be weighted because the difference appears to be very small. 

In our study population, VKAs were more often prescribed than DOACs overall. A Spanish study from between October 2015 and March 2016, including 837 patients with a mean age of 83.0 with 83.3% permanent AF, found that VKAs were the most common oral anticoagulants, prescribed in more than 2 out of 3 cases [24]. The persisting high prescription rate of VKAs is surprising because it is now acknowledged that, compared with VKAs, DOACs are associated with a reduced risk of stroke or systemic embolism, intracranial hemorrhage, and major bleeding in older persons with AF, even if they are associated with an increased risk of gastrointestinal bleeding [12,21]. Hence, in the absence of contraindications, the current guidelines recommend prescribing DOACs rather than VKAs for the management of AF [4]. The ongoing elevated rate of VKA prescriptions may be explained by a general reluctance to prescribe new drugs in the elderly, and physicians may prefer to wait until they have some experience with novel treatments in younger adults beforehand. Another possible explanation is the absence of biological tests to control the effectiveness of DOACs and to minimize the risk of overdose. Finally, the unavailability of a reversal agent for each DOAC could be a factor against prescribing these drugs. Indeed, at the time of the study, only dabigatran had a reversal agent, idarucizumab. These three explanations may also support the fact that people treated with DOACs had fewer RCDs and drugs per prescription than those treated with VKAs; DOACs were prescribed to older individuals considered to be the least physically frail. The daily cost of each of the two oral anticoagulants could explain the difference in prescribing rates, since DOAC treatment is 3 to 4 times more expensive than VKA treatment, including INR monitoring [25]. However, in France, when a disease is recognized as RCD, the resulting medical care, including prescriptions, are reimbursed in full. Seeing as our study focused on RCDs, including AF and VTE declared as such, the hypothesis according to which the physician would consider the cost remaining to be borne by the patient in the choice of the prescription of one or the other oral anticoagulant is unlikely. The publication of articles confirming that DOACs have a better benefit–risk ratio than VKAs or reporting data on efficacy and safety of DOACs [26] and increasing physician experience should progressively reverse prescribing trends, as should the arrival of andexanet alfa, the antidote of apixaban and rivaroxaban, which arrived on the market two years ago [27]. Concerning the mean number of drugs per prescription, it was higher in the VKA group than in the DOAC group and a factor associated with VKA prescription (in case of high mean number of drugs per prescription). This is potentially explained by the fact that subjects in the VKA group had more RCDs. However, the mean number of drugs per prescription was more than 5 in both groups. This confirms the polypharmacy trend in the older population. Drug–drug interactions and associated adverse events are frequent in individuals treated with several different molecules [28].

In our study, the fact that there were many more refill prescriptions (92.65% in DOAC group and 88.38% in VKA group) than novel prescriptions can be linked to the fact that GPs were by far the most frequent prescribers in our study population (90.70% in DOAC group and 94.71% in VKA group). Indeed, anticoagulants for AF or VTE, including pulmonary embolism, are very often initiated in a hospital and by a medical specialist and then renewed by the GP. This explanation may also apply to the fact that there were fewer initiations of DOACs than of VKAs (7.35% vs. 11.62%).

There were, significantly, more individuals in the VKA group with VTE, and multivariate analysis confirmed that VTE was an independent factor associated with VKA prescription. Here too, one of the possible explanations is the greater frailty of the population taking VKAs. Indeed, VTE increases the risk of frailty and poorer physical function [29]. However, bivariate analysis did not find a significant difference between the two groups for AF, and multivariate analysis found that AF was a determining factor for DOAC prescription. This is surprising given the direction of the other results of our study showing that frail people are more likely to be in the VKA group. Indeed, the risk factors for nonvalvular AF are common in frail people [30]. However, this result may reflect the beginning of a shift in the practices of GPs (the main prescribers in our study) towards the use of DOACs in the management of AF.

Severe heart failure or heart rhythm disorders were significantly less frequent in the DOAC group than in the VKA group (58.09% vs. 67.87%), and there was a similar result for severe hypertension (18.22% vs. 23.60%) and severe kidney diseases (1.49% vs. 3.82%). Multivariate analysis confirmed that these conditions were determining factors for VKA prescription. It is known that heart failure, hypertension and chronic kidney failure are associated with frailty [31,32,33], though only very few publications have examined the association between hypertension and frailty [34]. The fact that these factors (severe heart failure, hypertension and kidney diseases) were associated with the use of VKAs vs. DOACs could therefore be explained by their assimilation to frailty by the prescribers. In addition, the fact that severe renal failure was a factor associated with VKA prescription is an expected outcome considering that the variable degree of DOACs in kidney excretions (27%, 80%, 50%, and 33%, for apixaban, dabigatran, edoxaban, and rivaroxaban, respectively) could trigger a major risk of bleeding [14]. DOACs must be used with caution because a deficit in kidney excretions could lead to an increased bleeding risk in individuals with renal failure [14]. More specifically, a dose reduction is needed for all DOACs in case of moderate or severe renal failure, and dabigatran is strictly contraindicated when creatinine clearance is below 30 mL/min [35]. Thus, the French National Authority for Health (Haute Autorité de Santé (HAS)) recommends that VKAs be used rather than DOACs in cases of severe renal failure [36]. As with kidney failure, the association of heart rhythm disorders with the choice of VKAs rather than DOACs could be explained by the fact that the prescribers probably followed the indications. Indeed, VKAs are the only oral anticoagulants indicated in all heart rhythm disorders, including valvular AF.

Although our study included few individuals with active chronic liver diseases or cirrhosis (2 in each group), it is important to mention that, like for kidney failure, DOACs should be used cautiously in these individuals [37]. Apixaban and rivaroxaban are mainly eliminated by the liver (75% and 65%, respectively), edoxaban is half cleared by the liver, and dabigatran is predominantly eliminated by the kidney (only 20% by liver) [37]. In addition, plasma protein binding capacity varies according to DOACs. It is very high for rivaroxaban (95%) and apixaban (85%), moderate for edoxaban (55%), and lower for dabigatran (35%). This may result in higher free medication fraction levels when liver albumin synthesis is reduced [37]. Unlike for dabigatran and edoxaban, apixaban and rivaroxaban are mainly metabolized by cytochrome P450 enzymes, whose activity is diminished in case of liver disease [37]. Finally, the biliary excretion of all DOACs is altered in people with liver disease [37]. Nevertheless, studies showed that cirrhotic individuals taking DOACs rather than VKAs had similar risk of ischemic stroke, systemic embolism and intracranial bleeding [38], a lower risk of gastrointestinal bleeding [38], and comparable or lower risk of all-cause and major bleeding [39]. Thus, for these studies, DOACs are at least as effective and possibly safer than VKAs in case of chronic liver diseases or cirrhosis.

We found that disabling stroke, ischemic peripheral or coronary artery diseases, and diabetes, all of which are associated with frailty [32,40,41], were not predominant in either group.

Regarding concomitant cardiovascular drugs, angiotensin receptor blockers (10.79% vs. 13.97%), furosemide (40.81% vs. 49.66%) and digoxin (10.32% vs. 13.66%) were more commonly prescribed in the VKA group than in the DOAC group. Angiotensin receptor blockers were a determining factor for VKA prescription. This could be explained by the fact that VTE, severe heart failure, severe hypertension, and severe renal diseases were more common in the VKA group. However, the prescription rate of beta-blockers, calcium-channel-blockers, angiotensin-conversion-enzyme, thiazide diuretics, spironolactone, nitrate derivatives and hypolipidemic drugs were similar in the two groups. Furthermore, the presence of beta-blockers was a determining factor for DOAC prescription. This result seems consistent because AF was also found to be a determining factor for DOAC prescription, and beta-blockers are among the first-line drugs to control heart rate, especially in older persons [42,43].

Our study has some limitations. The first limitation is linked to the retrospective nature of the study. Indeed, this exposes our work to the same drawbacks as a real-world study, and the statistical significance of our results could be debatable. The fact that data are back-dated 3 years is also a limitation. This delay is in part explained by the duration of data extraction which was relatively long. The epidemiological situation may have changed since, according to the global trend towards the use of DOACs. Nevertheless, our study was performed around 10 years after the first DOAC (dabigatran) was marketed in France, and more than 5 years after the reimbursement approval of dabigatran for the treatment of NVAF. In our opinion, it is a fairly long time interval to assess prescription trends of oral anticoagulants. In that sense, our study remains interesting. The short timeframe (i.e., one month) of the study is also a limitation. Nevertheless, we included about 3200 individuals aged 80 years and older. Another limitation related to data collection is the fact that we did not have access to complete medical or socio-demographic records; hence the extrapolation of anticoagulant indication from RCDs. Additionally, some diseases considered non-serious by the physician may not have been recorded. This may have been the case for certain VTE events. Indeed, only VTE considered serious are declared as RCDs when the physician selects the category that specifies “non-exempting illness”. Meanwhile, AF is always declared as an RCD. The lack of access to complete medical or socio-demographic records also explains why we were unable to provide certain interesting data such as socioeconomic status, education levels, or estimated glomerular filtration rate. Because our work was based on regional data, results cannot be generalized to all types of populations. Nevertheless, this database remains very interesting because it can be used to produce relevant, real-life studies. Finally, it would be relevant to provide follow-up data for our population and to assess the impact of VKAs and DOACs after several years of treatment. However, the design of the present study did not include follow-up but was rather meant to provide a “snapshot” at a given time.

## 5. Conclusions

In our elderly population, DOACs were less prescribed than VKAs. Compared to patients prescribed DOACs, individuals prescribed VKAs were older and had more severe comorbidities (RCD), especially cardiovascular (except ischemic peripheral or coronary artery diseases) and severe chronic nephropathies, and more drugs per prescription. One possible explanation for the observed trend is the fact that some GPs, who were the main prescribers (>90%), may lack experience in prescribing DOACs. In addition, in clinical practice, physicians tend to be very cautious about prescribing new drugs in the elderly. We believe it would be worthwhile to conduct a similar study in the near future to assess whether the trend was reversed once GPs gained more experience and further robust research was published.

## Figures and Tables

**Table 1:** Comparison of age, sex, existence of one or more registered chronic diseases (RCD), anticoagulant prescription duration, medical specialty of the prescribing physician, rates of AF and VTE, mean number of RCDs, and mean number of drugs per prescription between subjects prescribed direct oral anticoagulants or vitamin K antagonists, using bivariate analysis by logistic regression.

Parameter		DOAC Group (*N* = 1279)	VKA Group (*N* = 1911)	OR (95% CI)	*p*
		Mean ± SD or % (N)	Mean ± SD or % (N)		
Mean age (years)		86.35 ± 4.29	87.11 ± 4.44	1.04 (1.02–1.06)	<0.001
Mean number of RCD		1.80 ± 1.17	2.07 ± 1.22	1.21 (1.13–1.29)	<0.001
Mean number of drugs/prescription		6.15 ± 2.84	6.66 ± 2.86	1.07 (1.04–1.09)	<0.001
Age range (years)	80–84	38.47 (492)	32.13 (614)	Reference	<0.001
	85–89	37.29 (477)	38.15 (729)	1.22 (1.04–1.44)	
	90–94	20.48 (262)	23.65 (452)	1.38 (1.14–1.68)	
	95–99	3.75 (48)	5.81 (111)	1.85 (1.29–2.65)	
	≥100	0.00 (0)	0.26 (5)	*	*
Sex	Women	51.92 (664)	48.25 (922)	Reference	0.043
	Men	48.08 (615)	51.75 (989)	1.16 (1.01–1.33)	
RCD	No RCD	10.40 (133)	7.27 (139)	Reference	0.002
	≥1 RCD	89.60 (1146)	92.73 (1772)	1.48 (1.15–1.90)	
Anticoagulant duration	Initiation	7.35 (94)	11.62 (222)	Reference	<0.001
	Refill	92.65 (1185)	88.38 (1689)	0.60 (0.47–0.78)	
Prescriber specialty	Other specialties	9.30 (119)	5.29 (101)	Reference	
	General practitioner	90.70 (1160)	94.71 (1810)	1.84 (1.40–2.42)	<0.001
Anticoagulation indication	Atrial fibrillation	41.36 (529)	44.22 (845)	1.12 (0.97–1.30)	0.117
	VTE	1.80 (23)	6.59 (126)	3.85 (2.46–6.05)	<0.001

DOAC: direct oral anticoagulant, VKA: vitamin K antagonist, N: number, OR: odds ratios, CI: confidence intervals, SD: standard deviation, RCD: registered chronic diseases, VTE: venous thromboembolism. * Insufficient number to calculate the OR and the *p* value.

**Table 2:** Comparison of registered chronic diseases and cardiovascular medications between subjects with direct oral anticoagulants and those with vitamin K antagonists, using bivariate analysis by logistic regression. Only significant differences are reported.

	DOAC Group (*N* = 1279)	VKA Group (*N* = 1911)	OR (95% CI)	*p*
	% (N)	% (N)		
RCD				
Severe heart failure or heart rhythm disorders	58.09 (743)	67.87 (1297)	1.52 (1.32–1.76)	<0.001
Severe hypertension	18.22 (233)	23.60 (451)	1.39 (1.16–1.66)	<0.001
Severe chronic respiratory failure	3.36 (43)	5.49 (105)	1.67 (1.16–2.40)	0.006
Severe chronic nephropathy and/or PNS	1.49 (19)	3.82 (73)	2.63 (1.58–4.39)	<0.001
Illnesses not on the list	9.07 (116)	13.61 (260)	1.58 (1.25–1.99)	<0.001
Drugs				
Angiotensin receptor blockers	10.79 (138)	13.97 (267)	1.34 (1.08–1.67)	0.009
Furosemide	40.81 (522)	49.66 (949)	1.43 (1.24–1.65)	<0.001
Digoxin	10.32 (132)	13.66 (261)	1.37 (1.10–1.72)	0.005
Other antiarrhythmic drugs	13.92 (178)	10.57 (202)	0.73 (0.59–0.91)	0.004

RCD: registered chronic diseases, DOAC: direct oral anticoagulant, VKA: vitamin K antagonist, N: number, OR: odds ratio, CI: confidence interval, PNS: primitive nephrotic syndrome.

**Table 3:** Comparison of selected parameters in subjects prescribed direct oral anticoagulants or vitamin K antagonists, using multivariate analyses by logistic regression. The results should be interpreted from the DOAC group.

Parameter	OR (95% CI)	*p*
Mean age (years)	0.96 (0.95–0.98)	<0.001
Mean number of drugs/prescription	0.94 (0.92–0.97)	<0.001
Male sex	0.79 (0.68–0.92)	0.002
Refill prescriptions	2.06 (1.56–2.71)	<0.001
General practitioner as prescriber	0.43 (0.31–0.58)	<0.001
Atrial fibrillation	1.23 (1.01–1.50)	0.045
VTE	0.23 (0.14–0.36)	<0.001
Severe heart failure or heart rhythm disorders	0.54 (0.44–0.66)	<0.001
Severe hypertension	0.82 (0.68–0.99)	0.036
Coronary artery disease	0.85 (0.69–1.04)	0.107
Severe chronic respiratory failure	0.72 (0.49–1.06)	0.095
Severe chronic nephropathy and/or PNS	0.39 (0.23–0.67)	<0.001
Beta-blockers	1.17 (1.01–1.37)	0.043
Angiotensin-receptor-blockers	0.76 (0.61–0.96)	0.021
Thiazide diuretics	1.39 (0.89–2.15)	0.143
Nitrate derivatives	1.39 (0.99–1.95)	0.051
Digoxin	0.79 (0.63–1.00)	0.052
Other antiarrhythmic drugs	1.38 (1.10–1.73)	0.006

OR: odds ratio, CI: confidence interval, VTE: venous thromboembolism, PNS: primitive nephrotic syndrome.

## Data Availability

The data presented in this study are available on request from the corresponding author. The data are not publicly available.

## References

[1] Romero-Ortuno R., Fouweather T., Jagger C. (2013). Cross-national Disparities in Sex Differences in Life Expectancy with and without Frailty. Age. Ageing..

[2] Izzo C., Carrizzo A., Alfano A., Virtuoso N., Capunzo M., Calabrese M., De Simone E., Sciarretta S., Frati G., Oliveti M. (2018). The Impact of Aging on Cardio and Cerebrovascular Diseases. Int. J. Mol. Sci..

[3] Béjot Y., Rouaud O., Jacquin A., Osseby G.V., Durier J., Manckoundia P., Pfitzenmeyer P., Moreau T., Giroud M. (2010). Stroke in the Very Old: Incidence, Risk Factors, Clinical Features, Outcomes and Access to Resources—A 22-year Population-based Study. Cerebrovasc. Dis..

[4] Hindricks G., Potpara T., Dagres N., Arbelo E., Bax J.J., Blomström-Lundqvist C., Boriani G., Castella M., Dan G.A., Dilaveris P.E. (2021). 2020 ESC Guidelines for the Diagnosis and Management of Atrial Fibrillation Developed in Collaboration with the European Association of Cardio-Thoracic Surgery (EACTS). Eur. Heart. J..

[5] Burnett A.E., Mahan C.E., Vazquez S.R., Oertel L.B., Garcia D.A., Ansell J. (2016). Guidance for the Practical Management of the Direct Oral Anticoagulants (DOACs) in VTE Treatment. J. Thromb. Thrombolysis..

[6] Symons G. (2014). Anticoagulation: Where Have We Come from and Where Are We Going? The Evidence for and against Novel Anticoagulants. South Afr. Med. J..

[7] Sommerauer C., Schlender L., Krause M., Weißbach S., Rieckert A., Martinez Y.V., Reeves D., Renom-Guiteras A., Kunnamo I., Sönnichsen A. (2017). Effectiveness and Safety of Vitamin K Antagonists and New Anticoagulants in the Prevention of Thromboembolism in Atrial Fibrillation in Older Adults–A Systematic Review of Reviews and the Development of Recommendations to Reduce Inappropriate Prescribing. BMC. Geriatr..

[8] Hoffman C., Leven C., Le Mao R., De Moreuil C., Lacut K. (2020). Anticoagulants Oraux Directs: Dans Quelles Indications? Lequel prescrire? Pour Ou Contre Chez les Personnes Fragiles et Dans les Situations Atypiques? Quelle Surveillance et Gestion des Accidents Hémorragiques?. Rev. Med. Interne..

[9] ANSM Bon Usage des Médicaments Antivitamine K (AVK). Actualisation–Juillet 2012..

[10] Salazar C.A., del Aguila D., Cordova E.G. (2014). Direct Thrombin Inhibitors versus Vitamin K Antagonists for Preventing Cerebral or Systemic Embolism in People with Non-valvular Atrial Fibrillation. Cochrane. Database. Syst. Rev..

[11] Sterne J.A., Bodalia P.N., Bryden P.A., Davies P.A., López-López J.A., Okoli G.N., Thom H.H., Caldwell D.M., Dias S., Eaton D. (2017). Oral Anticoagulants for Primary Prevention, Treatment and Secondary Prevention of Venous Thromboembolic Disease, and for Prevention of Stroke in Atrial Fibrillation: Systematic Review, Network Meta-analysis and Cost-effectiveness Analysis. Health. Technol. Assess..

[12] Ruff C.T., Giugliano R.P., Braunwald E., Hoffman E.B., Deenadayalu N., Ezekowitz M.D., Camm A.J., Weitz J.I., Lewis B.S., Parkhomenko A. (2014). Comparison of the Efficacy and Safety of New Oral Anticoagulants with Warfarin in Patients with Atrial Fibrillation: A Meta-analysis of Randomized Trials. Lancet.

[13] Almutairi A.R., Zhou L., Gellad W.F., Lee J.K., Slack M.K., Martin J.R., Lo-Ciganic W.H. (2017). Effectiveness and Safety of Non–vitamin K Antagonist Oral Anticoagulants for Atrial Fibrillation and Venous Thromboembolism: A Systematic Review and Meta-analyses. Clin. Ther..

[14] Lutz J., Jurk K., Schinzel H. (2017). Direct Oral Anticoagulants in Patients with Chronic Kidney Disease: Patient Selection and Special Considerations. Int. J. Nephrol. Renov. Dis..

[15] Steffel J., Verhamme P., Potpara T.S., Albaladejo P., Antz M., Desteghe L., Haeusler K.G., Oldgren J., Reinecke H., Roldan-Schilling V. (2018). The 2018 European Heart Rhythm Association Practical Guide on the Use of Non-vitamin K Antagonist Oral Anticoagulants in Patients with Atrial Fibrillation. Eur. Heart. J..

[16] ICD-10 Version: 2016. https://icd.who.int/browse10/2016/en.

[17] (2011). Décrets, arrêtés, circulaires. J. Off. De La République Française.

[18] Vaughan Williams E.M. (1992). Classifying Antiarrhythmic Actions: By Facts or Speculation. J. Clin. Pharmacol..

[19] (2019). R: A language and Environment for Statistical Computing. R Core Team.

[20] Le Bourg E. (2019). Is Life Expectancy of French Women Going to Plateau and Oscillate?. Gerontology.

[21] Shen N.N., Wu Y., Wang N., Kong L.C., Zhang C., Wang J.L., Gu Z.C., Chen J. (2020). Direct Oral Anticoagulants vs. Vitamin-k Antagonists in the Elderly with Atrial Fibrillation: A Systematic Review Comparing Benefits and Harms between Observational Studies and Randomized Controlled Trials. Front. Cardiovasc. Med..

[22] Wang N., Shen N.N., Wu Y., Zhang C., Pan M.M., Qian Y., Gu Z.C. (2020). Comparison of Effectiveness and Safety of Direct Oral Anticoagulants vs. Vitamin-k Antagonists in Elderly Patients with Atrial Fibrillation: A Systematic Review and Cost-effectiveness Analysis Protocol. Ann. Transl. Med..

[23] Sharma M., Cornelius V.R., Patel J.P., Davies J.G., Molokhia M. (2015). Efficacy and Harms of Direct Oral Anticoagulants in the Elderly for Stroke Prevention in Atrial Fibrillation and Secondary Prevention of Venous Thromboembolism: Systematic Review and Meta-analysis. Circulation.

[24] Mostaza J.M., Jiménez M.J.R., Laiglesia F.J.R., Peromingo J.A.D., Robles M.B., Sierra E.G., Bilbao A.S., Suárez C. (2018). Clinical Characteristics and Type of Antithrombotic Treatment in a Spanish Cohort of Elderly Patients with Atrial Fibrillation According to Dependency, Frailty and Cognitive Impairment. J. Geriatr. Cardiol..

[25] Base des Médicaments et Informations Tarifaires. Assurance Maladie. http://www.codage.ext.cnamts.fr/codif/bdm_it/index.php?p_site=AMELI.

[26] Caldeira D., Nunes-Ferreira A., Rodrigues R., Vicente E., Pinto F.J., Ferreira J.J. (2019). Non-vitamin K Antagonist Oral Anticoagulants in Elderly Patients with Atrial Fibrillation: A Systematic Review with Meta-analysis and Trial Sequential Analysis. Arch. Gerontol. Geriatr..

[27] Christensen H., Cordonnier C., Kõrv J., Lal A., Ovesen C., Purrucker J.C., Toni D., Steiner T. (2019). European Stroke Organisation Guideline on Reversal of Oral Anticoagulants in Acute Intracerebral Haemorrhage. Eur. Stroke. J..

[28] Gallo P., De Vincentis A., Pedone C., Nobili A., Tettamanti M., Gentilucci U.V., Picardi A., Mannucci P.M., Incalzi R.A., REPOSI Investigators (2019). Drug-drug Interactions Involving CYP3A4 and P-glycoprotein in Hospitalized Elderly Patients. Eur. J. Intern. Med..

[29] Lutsey P.L., Windham B.G., Misialek J.R., Cushman M., Kucharska-Newton A., Basu S., Folsom A.R. (2020). Long-term Association of Venous Thromboembolism with Frailty, Physical Functioning, and Quality of Life: The Atherosclerosis Risk in Communities Study. J. Am. Heart. Assoc..

[30] Wasmer K., Eckardt L., Breithardt G. (2017). Predisposing Factors for Atrial Fibrillation in the Elderly. J. Geriatr. Cardiol..

[31] Vitale C., Uchmanowicz I. (2019). Frailty in Patients with Heart Failure. Eur. Heart. J. Suppl..

[32] Castrejón-Pérez R.C., Gutiérrez-Robledo L.M., Cesari M., Pérez-Zepeda M.U. (2017). Diabetes Mellitus, Hypertension and Frailty: A Population-based, Cross-sectional Study of Mexican Older Adults. Geriatr. Gerontol. Int..

[33] Walker S.R., Gill K., Macdonald K., Komenda P., Rigatto C., Sood M.M., Bohm C.J., Storsley L.J., Tangri N. (2013). Association of Frailty and Physical Function in Patients with Non-dialysis CKD: A Systematic Review. BMC Nephrol..

[34] Fabbian F., De Giorgi A., Cappadona R., Gozzi D., Pasin M., De Giorgio R., Manfredini R. (2018). Hypertension, Abnormal Blood Pressure Circadian Pattern, and Frailty: Data from the Literature. J. Geriatr. Cardiol..

[35] Parker K., Thachil J. (2018). The Use of Direct Oral Anticoagulants in Chronic Kidney Disease. Br. J. Haematol..

[36] Fibrillation Auriculaire non Valvulaire. Quelle Place pour les Anticoagulants Oraux non Antivitamine K: Apixaban (Eliquis^®^), Dabigatran (Pradaxa^®^) et Rivaroxaban (Xarelto^®^). HAS. Bon Usage du Médicament. https://www.has-sante.fr/upload/docs/application/pdf/2013-07/fs_bum_naco_v5.pdf.

[37] Ballestri S., Capitelli M., Fontana M.C., Arioli D., Romagnoli E., Graziosi C., Lonardo A., Marietta M., Dentali F., Cioni G. (2020). Direct Oral Anticoagulants in Patients with Liver Disease in the Era of Non-Alcoholic Fatty Liver Disease Global Epidemic: A Narrative Review. Adv. Ther..

[38] Lee H.F., Chan Y.H., Chang S.H., Tu H.T., Chen S.W., Yeh Y.H., Wu L.S., Kuo C.F., Kuo C.T., See L.C. (2019). Effectiveness and Safety of Non-vitamin K Antagonist Oral Anticoagulant and Warfarin in Cirrhotic Patients with Nonvalvular Atrial Fibrillation. J. Am. Heart. Assoc..

[39] Lapumnuaypol K., Di Maria C., Chiasakul T. (2019). Safety of Direct Oral Anticoagulants in Patients with Cirrhosis: A Systematic Review and Meta-analysis. QJM.

[40] Woods N.F., LaCroix A.Z., Gray S.L., Aragaki A., Cochrane B.B., Brunner R.L., Masaki K., Murray A., Newman A.B., Women’s Health Initiative (2005). Frailty: Emergence and Consequences in Women Aged 65 and Older in the Women’s Health Initiative Observational Study. J. Am. Geriatr. Soc..

[41] Lin C.H., Chou C.Y., Liu C.S., Huang C.Y., Li T.C., Lin C.C. (2015). Association between Frailty and Subclinical Peripheral Vascular Disease in a Community-dwelling Geriatric Population: Taichung Community Health Study for Elders. Geriatr. Gerontol. Int..

[42] Van Gelder I.C., Rienstra M., Crijns H.J., Olshansky B. (2016). Rate Control in Atrial Fibrillation. Lancet.

[43] (2014). Guide Parcours de Soins Fibrillation Atriale.

